# Marker-assisted selection of YY supermales from a genetically improved farmed tilapia-derived strain

**DOI:** 10.24272/j.issn.2095-8137.2018.071

**Published:** 2018-09-06

**Authors:** Chao-Hao Chen, Bi-Jun Li, Xiao-Hui Gu, Hao-Ran Lin, Jun-Hong Xia

**Affiliations:** 1 State Key Laboratory of Biocontrol, Institute of Aquatic Economic Animals and Guangdong Provincial Key Laboratory for Aquatic Economic Animals, College of Life Sciences, Sun Yat-Sen University, Guangzhou Guangdong 510275, China

**DEAR EDITOR**,

Genetically improved farmed tilapia (GIFT) and GIFT-derived strains account for the majority of farmed tilapia worldwide. As male tilapias grow much faster than females, they are often considered more desirable in the aquacultural industry. Sex reversal of females to males using the male sex hormone 17-α-methyltestosterone (MT) is generally used to induce phenotypic males during large-scale production of all male fingerlings. However, the widespread use of large quantities of sex reversal hormone in hatcheries may pose a health risk to workers and ecological threats to surrounding environments. Breeding procedures to produce genetically all-male tilapia with limited or no use of sex hormones are therefore urgently needed. In this study, by applying marker-assisted selection (MAS) for the selection of YY supermales from a GIFT-derived strain, we identified 24 XY pseudofemale and 431 YY supermale tilapias. Further performance evaluation on the progenies of the YY supermales resulted in male rates of 94.1%, 99.5% and 99.6%, respectively, in three populations, and a daily increase in body weight of 1.4 g at 3 months (*n*=997). Our study established a highly effective MAS procedure in the selection of YY supermales from a GIFT-derived strain. Furthermore, the development of MAS-selected YY supermales will help reduce the utilization of hormones for controlling sex in the tilapia aquaculture.

Tilapia is the second most important fish species in the global aquaculture industry after carp (FAO, 2010). China is the world’s largest tilapia producer, with total production of 1.87 million metric tons (MMT) in 2016, and is expected to exceed 1.9 MMT in 2017 (Bureau of Fisheries of the Ministry of Agriculture, 2018). Total tilapia exports could reach 875 000 tons, accounting for 45% of total tilapia production in 2017 (Bureau of Fisheries of the Ministry of Agriculture, 2018).

Several species of tilapia (such as Nile tilapia, *Oreochromis niloticus*, blue tilapia, *Oreochromis aureus* and Mozambique tilapia, *Oreochromis mossambicus*) are cultured commercially. Genetically improved farmed tilapia (GIFT) and GIFT-derived strains are the predominant cultured tilapia strains worldwide due to their faster growth rates, higher survival rates, shorter harvest time, and dramatically increased yields (ADB, 2005; Van Bers et al., 2012).

Male GIFT grow much faster than females. In mixed-sexed populations, uncontrolled reproduction leads to excessive recruitment of fingerlings, competition for food, and stunting of the original stock. Therefore, sex reversal of females to males is desirable in tilapia aquaculture. The male sex hormone 17-α-methyltestosterone (MT) is often used to induce phenotypic males. However, widespread use of large quantities of sex reversal hormone in hatcheries may pose a health risk to workers (Mair, 1997) and an ecological threat to the surrounding environment. Therefore, breeding procedures to produce genetically all-male tilapia with lower or no use of sex hormone are urgently needed.

*Tilapia mossambica* YY supermales were originally produced by crossing sex-reversed XY pseudofemales obtained by hormone treatment with normal XY males (Mair, 1988, 1997), with the supermales then identified by progeny testing. However, this breeding process was highly time-consuming. In contrast, marker-assisted selection (MAS) can greatly improve selection efficiency compared with traditional methods using markers closely linked to economic traits (Rishell, 1997). Sex-linked markers in tilapia have been identified on LG1 (Palaiokostas et al., 2013), LG3 (Cnaani & Kocher, 2008), LG8 (Lee et al., 2003), LG22 (Liu et al., 2013) and LG23 (Eshel et al., 2011; Sun et al., 2014), thus providing a solid basis for applying MAS in the sex control of tilapia (Chen et al., 2018). YY supermales from Nile tilapia have been identified using sex-linked markers, with the YY progeny reported to be 100% male (Li & Wang, 2017). These suggests it is possible to produce YY supermales by MAS in tilapia breeding.

GIFT and GIFT-derived strains are the most commonly farmed tilapia in the world, with much higher market value than unselected Nile tilapia (Bureau of Fisheries of the Ministry of Agriculture, 2018; ADB, 2005). However, no GIFT-derived strain fingerlings selected using MAS are currently available in the seed market. In this study, we reported on the selection of more than 400 YY supermales from a GIFT-derived strain using MAS. The new GIFT-derived YY supermale populations with high male offspring rates will be helpful in controlling or limiting the use of sex hormones in tilapia aquaculture.

This study was approved by the Animal Care and Use Committee from the School of Life Science at Sun Yat-Sen University.

In the hatchery, we first selected 20 large males and 20 large females from the broodstock population of nearly 500 individuals from Wulonggang Aquaculture Ltd., Guangzhou, Guangdong Province, China. One male (with red flush to head, lower body, dorsal and caudal fins) and one female (ready to spawn, showing pink to red and protruding genital papilla, fully opened genital pore, and distended abdomen) were then selected based on body size, body shape and reproductive status. One full-sib family (named WLG-Fam1-F1) was established by crossing these two individuals. The swim-up fry were divided into three experimental groups of ~500 individuals and reared in independent nursery tanks (5 m×5 m×1 m). The fry from one tank were fed normal food. The fry from the other two tanks (for sex-reversal treatment) were provided with feed treated with diethylstilbestrol (DES, Aladdin, China) at 0.5 g or 1 g per kg of feed. The DES was first dissolved with ethanol to prepare a stock solution, which was then thoroughly mixed with the feed. The dried pellets were provided to the fry twice a day from 3 d post hatchery (dph) for three weeks.

At 30 dph, the fingerlings from each group were transferred to a pond (around 1 000 m^2^), respectively. The fish were fed twice daily with commercial pellets. At 120 dph, the sex phenotype and body weight of each fish was recorded, and fin samples from each fish were also collected and kept in absolute ethanol for DNA extraction and genotyping.

We used the SD marker closely linked to sex trait located on chrLG23 (forward primer: 5′-TCCCATTTAGACC ACCACACCTCAACAACA-3′; reverse primer: 5′-GTCAGAATGCACTTTAACACAGAGATACCA-3′; patent application no.: 2016107162044) to genotype each individual. This marker was determined to be family-specific as it was not significantly associated with sex traits in another full-sib tilapia family in our pilot study. PCR amplifications were performed as per previous research (Gu et al., 2018) and were carried out in a 20-µL volume using 2× PCR mastermix, 1 ng genomic DNA, and 0.5 µmol/L forward and reverse primers (Dongsheng, China) in a BioRad cycler. The following program was applied (one cycle of 3 min at 94 °C, 38 cycles of 30 s at 94 °C, 30 s at 55 °C and 30 s at 72 °C, followed by a final extension of 5 min at 72 °C). The resulting PCR products were detected by electrophoresis with 6% polyacrylamide gels and silver staining. The pseudofemales (XY genotype), XX females and XY males were identified based on phenotypic sex traits and genotypic data.

We generated F2 families by crossing six pseudofemales (XY genotype) with three genetic males (XY genotype). At 120 dph, the sex of each fish was recorded, and fin samples were collected and kept in absolute ethanol for DNA extraction and genotyping. The supermales (YY genotype) were identified based on phenotypic sex traits and genotypic data of the SD marker.

Two YY supermales were randomly selected for crossing with three XX GIFT-derived females collected from the same farm. At 21 dph, around 2 000 fingerlings (0.7 g) were transferred into a pond (1 000 m^2^) for maturation. The fish were fed twice daily with commercial pellets. At 89 dph, the sex of each fish was recorded, and fin samples were collected and kept in absolute ethanol for DNA extraction and genotyping. We also recorded the body weights of 61 randomly selected individuals with a weighing machine.

To test the rate of male offspring, two other populations were also generated by crossing two randomly selected YY supermales with three GIFT-derived females collected from other farms or with three GIFT-derived females collected from the same family that generated YY supermales. The sex of each fish was manually checked at 240 dph.

We harvested 1 356 F1 generation individuals (WLG-Fam1-F1) from the three groups (Table 1). In group 1 (treated with 1 g DES/kg feed), all individuals (*n*=330) were female (100%). In group 2 (treated with 0.5 g DES/kg feed), we found 371 of the 390 individuals were phenotypically female (95%). In contrast, the female rate (*n*=636) was only 30% in the control group (no DES treatment). These data suggest the DES treatment was highly effective for sex reversal in this study.

**Table 1:** Summary information on sex reversal of the F1 generation

Group	DES (g)/feed (kg)	Individual number (*n*)	Female	Male	Intersex	Female rate (%)
Group1	1	330	330	0	0	100
Group2	0.5	390	371	6	13	95
Control	No DES	636	190	446	0	30
Total	N/A	1 356	891	452	13	N/A

N/A: Not available.

**Figure 1:**
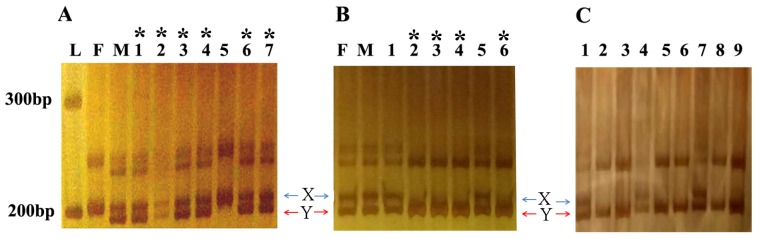
Genotyping of individuals during marker-assisted selection using the sex-linked marker SD

Based on the sex phenotype and body weight (data not provided), 73 faster-growing females from WLG-Fam1-F1 were selected for genotyping using the SD marker closely linked to sex trait previously developed in our lab. From this female population, 24 XY pseudofemales were finally identified. Genotyping of the female population is shown in Figure 1A.

A: Screening of pseudofemales. L: ladder; “F” and “M” indicate female and male parents, respectively; lanes 1 to 7 indicate seven randomly selected female individuals from WLG-F1-F1; band “Y” is the male-specific band and band “X” is the female band. A symbol “*” indicates pseudofemale detected in the F1 population. B: Screening of YY supermales. “F” and “M” indicate XY pseudofemale and XY male parents, respectively; lanes 1 to 6 indicate six randomly selected male individuals from WLG-F1-F2; band “Y” is male-specific band and band “X” is female band. A symbol “*” indicates YY supermales identified in the F2 population. C: Genotyping analysis of all-male offspring produced using YY supermales with XX females. Lanes 1 to 9 indicate nine randomly selected all-male offspring from WLG-F1-F3; band “Y” is male-specific band.

Six XY pseudofemales were then crossed with three XY males to generate the F2 generation (WLG-F1-F2). At 120 dph, we harvested 1 770 adult male tilapias from the WLG-Fam1-F2 population. Genotypic analysis using the SD marker indicated that 431 males had the YY genotype and the remaining males had the XY genotype. Genotyping of the WLG-F1-F2 population is shown in Figure 1B. Surprisingly, the YY rate in the male population was around 25%, which is lower than the expected rate (~33%). In addition, we randomly measured the body weights of 40 individuals with the YY, XY and XX genotypes, respectively. The average body weight for the YY supermales was the lowest (average 69.5±23.89 g), followed by the XX females (77.1±38.07 g) and normal XY males (94.6±28.24 g). These data indicate that several unknown factors have affected the survivability and growth of YY supermales in this study. However, additional data from different families and populations are required to clarify this in future research.

Two randomly selected YY supermales were crossed with five normal XX females collected from local farms to generate the F3 generation (WLG-Fam1-F3). At 89 dph, 994 of the 997 harvested individuals were phenotypically male and three were phenotypically female. Thus, the male rate was 99.6% in the progeny population of the YY supermales.

**Figure 2:**
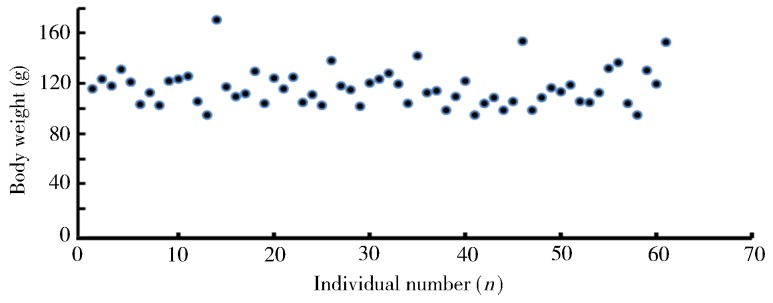
Distribution of body weight (g) among 61 all-male offspring from WLG-Fam1-F3 at 89 dph

We randomly collected fin samples from 61 phenotypic males for genotyping with the SD marker. Results demonstrated that all phenotypic male progenies possessed a male-specific band ‘Y’ (Figure 1C), thus indicating the reliability of the GIFT-derived strain YY supermales in this study. The average body weight for the 61 individuals was 117±14.9 g (Figure 2) and the daily increase in body weight was around 1.4 g. These data are comparable to the values reported in previous studies; for example, the daily increase in body weight at 3 months for a GIFT strain in Guangxi Province, China was reported to be 1.4–1.6 g (Tand et al., 2011). Thus, in the current study, the F3 all-male offspring grew as quickly as the normal GIFT strain and the differences among individual body weights were small, which would be advantageous for tilapia aquaculture.

To test the reliability of the YY supermales, two other populations were constructed by crossing YY supermales with females collected from other farms or from the same family generating YY supermales. We observed a male rate of 99.5% for the population (*n*=200) generated using females from other farms and 94.1% for the population (*n*=187) generated using females from the same family as YY supermales. Thus, these results again show that the offspring developed here are highly suitable for tilapia aquaculture.

The threat of environmental hormones has emerged as a worldwide concern, and can disrupt the central nervous system, pituitary integration of hormonal and sexual behavior, female and male reproductive system development and function, and thyroid function (Crisp et al., 1998). MT is a synthetic male hormone widely used in animal products to promote weight gain. There are no reports showing that MT treatment of tilapia affects human health, provided it is applied only during the early fry stage at the recommended dosage. However, widespread use of large quantities of sex reversal hormones in hatcheries may pose a health risk to workers (Mair, 1997) and cause potential hormone contamination to surrounding environments due to the release of wastewater from tilapia production facilities that utilize MT treatment. Sex control for the production of all-male tilapia in breeding procedures is considered to be a potential solution to the problem.

The GIFT program successfully resulted in an accumulated response of 85% after six generations of selection (Eknath et al., 1998). Currently, more than 100 countries now farm tilapia (FAO, 2013), with GIFT or GIFT-derived strains the major cultured tilapia strains worldwide. For example, in the Philippines, 70% of farmed tilapia are either GIFT or of GIFT-derived origin (ADB, 2005).

Previous studies have shown that GIFT or GIFT-derived strains possess diverse genetic sources that are different from the Nile tilapia. Founders of GIFT strains are composed of eight different populations of Nile tilapia (four wild tilapia populations from Egypt, Ghana, Kenya, and Senegal and four farmed populations from Israel, Singapore, Taiwan, and Thailand) (Van Bers et al., 2012). Genetic analysis has shown that the development of many GIFT or GIFT-derived strains has led to the coexistence of different varieties and levels of hybridization among stocks (McKinna et al., 2010; Xia et al., 2014). Previous mtDNA analysis of a Fijian GIFT strain and WorldFish Centre strain revealed introgression of two *Oreochromis* species (*O. mossambicus* and *O. aureus*) to the GIFT strain, which is considered to be an improved line to the Nile tilapia (McKinna et al., 2010). Our previous study also suggested slight introgression from Mozambique tilapia to the GIFT strain, with GIFT and Nile tilapia significantly and genetically differentiated from red tilapia and Mozambique tilapia (Xia et al., 2014). These findings provide good explanation for their differences in economic traits.

Efforts to select YY supermale tilapia have been conducted previously (Li & Wang, 2017; Yang et al., 1980). However, the commercial production of MAS-selected all-male tilapia is not available currently in China. In our study, we focused on the MAS of YY supermales from a GIFT-derived strain. Using a sex-linked marker, we selected 24 pseudofemales (XY genotype) from 73 females and 431 supermales (YY genotype) from 1 770 adult males from the WLG-F1-F2 population. From the three offspring populations of the YY supermales, male offspring rates were 94.1%, 99.5% and 99.6%, respectively. Previous studies on tilapia have shown that the mean percentage of males obtained by MT treatment is 68% to 99% (Kohinoor et al., 2003; Shamsuddin et al., 2012), whereas YY progenies are 100% male in MAS selection (Li & Wang, 2017). Therefore, the male rates observed in our study are comparable to the results of former studies. Furthermore, we observed a daily increase in body weight of around 1.4 g, which is also comparable to the values reported in previous studies. For example, the daily increase in body weight at 3 months for a GIFT strain from Guangxi Province, China, was reported to be 1.4–1.6 g (Tand et al., 2011). Thus, our study indicates the YY supermales are suitable for production of aquaculture seeds.

However, in the control group identifying pseudofemales, we observed that ~30% of the progeny were females. Thus, the sex segregation ratio of female:male was ~3:7. This suggests a male priority for sex determination/differentiation under the environmental conditions. Temperature is one of the most important environmental factors that influence sexual determination in tilapia. Although low temperature treatment (18 °C) is not generally effective at influencing sex ratios (Tessema et al., 2006), previous research has shown that at high temperatures, 91.2% of progeny exhibited an excess of males in three populations of Nile tilapia (Bezault et al., 2007). Another study further indicated that when the water temperature increased from 25 °C to 35 °C, the male ratio significantly increased from 52.47% to 75.91% after one week of rearing (Khater et al., 2017). In the current study, progenies were raised in outdoor tanks and ponds during early development, without water temperature control. Therefore, in the field test of YY progenies, the male rates of 94%–99.6% were likely to be overestimated due to the temperature effect on the sex determination. Nevertheless, it has been suggested that thermal effects are secondary to genetics in zebrafish, as sex ratio only changes at extreme water temperatures (Ospina-Álvarez & Piferrer, 2008). To clearly address the environmental effects on the male offspring rate, a control group under the same environmental conditions should be established in the future.

MAS has only been applied in very limited aquaculture species, such as selection of resistance to lymphocystis disease in Japanese flounders and resistance to infectious pancreatic necrosis (IPN) in salmon (Yue, 2014). Our study, as well as that of Li & Wang (2017), indicate that MAS can be highly valuable for sex control in tilapia. Currently, in addition to sex determination traits (Eshel et al., 2011; Lee et al., 2003, 2004; Shirak et al., 2006), many quantitative trait loci controlling other economically beneficial traits have been mapped for tilapia, e.g., body size (Cnaani et al., 2004), temperature tolerance (Cnaani et al., 2003), general disease resistance and immune response (Cnaani et al., 2004), body color (Lee et al., 2005), salinity tolerance (Gu et al., 2018; Rengmark et al., 2007) and hypoxia stress (Li et al., 2017). These data provide a cost-effective starting point to search for markers closely linked to economic traits within breeding populations. Therefore, we would expect greater application of MAS in commercial breeding programs in the tilapia aquaculture industry in the future.

By applying MAS for the selection of supermale GIFT-derived strains, we established a set of MAS procedures and obtained more than 400 YY supermales. Analysis of the progeny rates indicated the reliability of the YY supermales. Further systematic evaluation of the mating strategies with different sources of females will help speed up the commercial production of all-male seeds.
